# Supplementary material for: Differential Expression of Amanitin Biosynthetic Genes and Novel Cyclic Peptides in Amanita molliuscula
Source: J Fungi (Basel). 2021 May 14;7(5):384. doi: 10.3390/jof7050384 (PMC8156247; doi:10.3390/jof7050384)
Supplement: Supplementary file 1 [file jof-07-00384-s001.zip › supplementary files/Supplementary file 1.docx]

Supplementary file 1: The gene information for phylogenetic reconstruction.

>Amanita_franzii HKAS77321 (MH508357)

GGATCATTAATGAATGAACCTGAGGCTGTTGCTGGCCCCTGAAAAGGGGGGCATGTGCACGTCTCTATTCATCACCCATTCCACCTGTGCACTACTGTAGACACCTGGGAATGAGAGACCTTCATTGGGCTCTTGGGCTTATTGAAATCTGGGTGTCTATGATATTTGTATGAAACACTGTTTGGCATGAATGATAAAAAGTAGGGCTTTTTGCCTGTAATTAAAGTACAACTTTCAACAACGGATCTCTTGGCTCTCGCATCGATGAAGAACGCAGCGAAATGCGATAAGTAATGTGAATTGCAGAATTCAGTGAATCATCGAATCTTTGAACGCACCTTGCGCTCCTTGGTATTCCAAGGAGCATGCCTGTTTGAGTGTCATTAATATCTCAAGATGCATCATCTGTTGTATGATACTCTTGGATGTTGGGGGTTGCAGGCTTGAGAAAAAAGCCAGCTCCCCTTGAATGTATTAGTGGAGAGGACAATTGAACTCCATGGTGTGATAAAATCTGTCTATGCCAGGAGTATGTTTGGCTCTCTGTTACCAAACTGTCCAGAATGGACAACTTGATCAACTTGACCTCAA

>Amanita_franzii HKAS91231 (MH508358)

GGATCATTAATGAATGAACCTGAGGCTGTTGCTGGCCCCTGAAAAAAAGGGGGGCATGTGCACGTCTCTATTCATCACCCATTCCACCTGTGCACTACTGTAGACACCTGGGAATGAGAGACCTTCATTGGGCTCTTGGGCTTATTGAAATCTGGGTGTCTATGATATTTGTATGAAACACTGTTTGGCATGAATGATAAAAAGTAGGGCTTTTTGCCTGTAATTAAAGTACAACTTTCAACAACGGATCTCTTGGCTCTCGCATCGATGAAGAACGCAGCGAAATGCGATAAGTAATGTGAATTGCAGAATTCAGTGAATCATCGAATCTTTGAACGCACCTTGCGCTCCTTGGTATTCCAAGGAGCATGCCTGTTTGAGTGTCATTAATATCTCAAGATGCATCATCTGTTGTATGATACTCTTGGATGTTGGGGGTTGCAGGCTTGAGAAAAAAGCCAGCTCCCCTTGAATGTATTAGTGGAGAGGACAATTGAACTCCATGGTGTGATAAAATCTGTCTATGCCAGGAGTATGTTTGGCTCTCTGTTACCAAACTGTCCAGAATGGACAACTTGATCAA

>Amanita_zangii GDGM29241 (KJ466432)

GGATCATTAATGAATGCACTTATGAGACTGTTGCTGGTCCTTGGGGGCATGTGCACGTCTCAAAGTCATTACCAATTCCACCTGTGCACAAGTGTAGACACTCAGGAATGTCTTGGTGTCTATGTCATTTCATTAAACACAATTGTATGTCTATAGAATGGATGTCATGCATTAAAGTACAACTTTCAACAACGGATCTCTTGGCTCTCGCATCGATGAAGAACGCAGCGAAATGCGATAAGTAATGTGAATTGCAGAATTCAGTGAATCATCGAATCTTTGAACGCACCTTGCGCTCCTTGGTATTCCGAGGAGCATGCCTGTTTGAGTGTCATTAAAATCTCAAGACCCCCATCTGCTTTGGATGGGGTTATTGGATGGCTGGGGGTTGCAGGCTTTTCAATAGCCAGCTCTCCTTGAATGAATTAGTGGGGAAGACAATTGAACTCCATTGGTGTGATAAATTATCTATGCCAGGAGCAGGATTGGGTCTACTGCTGTCTAACTGTCCAGAAAAGAGGACAAAAACAAACTTGACCTCAA

>Amanita_zangii HKAS99663 (MH508655)

GGATCATTAATGAATGCACTTCTGAGACTGTTGCTGGTCCTTGGGGGCATGTGCACGTCTCAAAGTCATTACCAATTCCACCTGTGCACAAGTGTAGACACTCAGGAATGTCTTGGTGTCTATGTCATTTCATTAAACACAATTGTATGTCTATAGAATGGATGTCATGCATTAAAGTACAACTTTCAACAACGGATCTCTTGGCTCTCGCATCGATGAAGAACGCAGCGAAATGCGATAAGTAATGTGAATTGCAGAATTCAGTGAATCATCGAATCTTTGAACGCACCTTGCGCTCCTTGGTATTCCGAGGAGCATGCCTGTTTGAGTGTCATTAAAATCTCAAGACCCCCATCTGCTTTGGATGGGGTTATTGGATGGCTGGGGGTTGCAGGCTTTTCAATAGCCAGCTCTCCTTGAATGAATTAGTGGGGAAGACAATTGAACTCCATTGGTGTGATAAATTATCTATGCCAGGAGCAGGATTGGGTCTCTGCTGTCTAACTGTCCAGAAAAGAGGACAAAAACAATACTTGACCTCAA

>Amanita_exitialis HKAS74673 (KJ466375)

GGATCATTAATGAAATGAATCTTGAGGCTGTCGCTGGCCCATCTGGGCATGTGCACGTCTCTGGTCATTACCAATTCCACCTGTGCACACTTGTAGACACTTGGGAATGAGAGGCTTTGACCAGTCTCTTGAAGTTGAAATCTGGGTGTCTATGGCATTTTATTAAACACTAGTTGCATGTTTATAGAATGATGATTTGAATATATATATATAAAGTACAACTTTCAACAACGGATCTCTTGGCTCTCGCATCGATGAAGAACGCAGCGAAATGCGATAAGTAATGTGAATTGCAGAATTCAGTGAATCATCGAATCTTTGAACGCACCTTGCGCTCCTTGGCATTCCAAGGAGCATGCCTGTTTGAGTGTCATTAAAGTCTCAAGACCTGTCTGATTTTGATAGGTATTGGATTTTGGGGGTTGCAGGCTTTTTCAGACCGCCTGCTCTCCTTGAATGTATTAGTGGAGAAAAAGCCATTTGAACTCCATTGGTGTGATAAAATCTATCAATGCCAGGAGCAATGCTAGTAATCTCTGCTGTCTAACTGTCTGTAAAAATGGACAATTTGACCAACTTGACCTCAA

>Amanita_exitialis HKAS75774 (JX998027)

GGATCATTAATGAAATGAATCTTGAGGCTGTCGCTGGCCCATCTGGGCATGTGCACGTCTCTGGTCATTACCAATTCCACCTGTGCACACTTGTAGACACTTGGGAATGAGAGGCTTTGACCAGTCTCTTGAGAAGTTGAAATCTGGGTGTCTATGGCATTTTATTAAACACTAGTTGCATGTTTATAGAATGATGATTTGAATATATATATAAAGTACAACTTTCAACAACGGATCTCTTGGCTCTCGCATCGATGAAGAACGCAGCGAAATGCGATAAGTAATGTGAATTGCAGAATTCAGTGAATCATCGAATCTTTGAACGCACCTTGCGCTCCTTGGCATTCCAAGGAGCATGCCTGTTTGAGTGTCATTAAAGTCTCAAGACCTGTCTGATTTTGATAGGTATTGGATTTTGGGGGTTGCAGGCTTTTTCAGACTGCCTGCTCTCCTTGAATGTATTAGTGGAGAAAAAGCCATTTGAACTCCATTGGTGTGATAAAATCTATCAATGCCAGGAGCAATGCTAGTTATCTCTGCTGTCTAACTGTCTGTAAAAATGGACAATTTGACCAACTTGACCTCAAATCAGGTAGGACTACCCGCTGA

>Amanita_exitialis HKAS75775 (JX998026)

GGATCATTAATGAAATGAATCTTGAGGCTGTCGCTGGCCCATCTGGGCATGTGCACGTCTCTGGTCATTACCAATTCCACCTGTGCACACTTGTAGACACTTGGGAATGAGAGGCTTTGACCAGTCTCTTGAGAAGTTGAAATCTGGGTGTCTATGGCATTTTATTAAACACTAGTTGCATGTTTATAGAATGATGATTTGAATATATATATAAAGTACAACTTTCAACAACGGATCTCTTGGCTCTCGCATCGATGAAGAACGCAGCGAAATGCGATAAGTAATGTGAATTGCAGAATTCAGTGAATCATCGAATCTTTGAACGCACCTTGCGCTCCTTGGCATTCCAAGGAGCATGCCTGTTTGAGTGTCATTAAAGTCTCAAGACCTGTCTGATTTTGATAGGTATTGGATTTTGGGGGTTGCAGGCTTTTTCAGACTGCCTGCTCTCCTTGAATGTATTAGTGGAGAAAAAGCCATTTGAACTCCATTGGTGTGATAAAATCTATCAATGCCAGGAGCAATGCTAGTTATCTCTGCTGTCTAACTGTCTGTAAAAATGGACAATTTGACCAACTTGACCTCAAATCAGGTAGGACTACCCGCTGA

>Amanita_fuliginea HKAS100628 (MH508369)

GGATCATTAAAGAAATGAACCTTGAGACTGTCGCTGGCCCCTCCTGCAGATGGAGTTGGCATGTGCACGTCTCTGGTCATTACCAATTCCACCTGTGCACACTTGTAGACACTTGGGAATGAGAGACATAGGCTGGTTGGCTGGTCTCTTGAGAGAGAGAAATGAAAAAAATCTGGGTGTCTATGCCATTTTTATTAAACACTAGTTGCATGTTTATAGAATGAATGATGATTTGATTGAATATGAAATACAACTTTCAACAACGGATCTCTTGGCTCTCGCATCGATGAAGAACGCAGCGAAATGCGATAAGTAATGTGAATTGCAGAATTCAGTGAATCATCGAATCTTTGAACGCACCTTGCGCTCCTTGGCATTCTGAGGAGCATGCCTGTTTGAGTGTCATTAACATCTCAATACCTGTCTGCTTTTGATGGGTATTGGAAATTGGGGGTTGCAGGGCTTGTATAGCTTTGCTCTCCTTGAATGTATTAGTGGAGAAAAAGAAAGCTCATTGAACTCCATTGGTGTGATAAAATCTATCAATACCAGGAACAATGTCCAAGTTTCTCTGCTCTCTAATCCATGTCCAGTTTGGACCTCAT

>Amanita_fuliginea HKAS75780 (JX998023)

GGATCATTAAAGAAATGAACCTTGAGACTGTCGCTGGCCCCTCCTGCAGATGGAGTTGGCATGTGCACGTCTCTGGTCATTACCAATTCCACCTGTGCACAATTGTAGACACTTGGGAATGAGAGACATAGGCTGCTTGGCTGGTCTCTTGAGAGAGAGAAATGGAAAAAATCTGGGTGTCTATGCCATTTTTATTAAACACTAGTTGCATGTTTATAGAATGAATGATGATTTGATTGAATATGAAATACAACTTTCAACAACGGATCTCTTGGCTCTCGCATCGATGAAGAACGCAGCGAAATGCGATAAGTAATGTGAATTGCAGAATTCAGTGAATCATCGAATCTTTGAACGCACCTTGCGCTCCTTGGCATTCTGAGGAGCATGCCTGTTTGAGTGTCATTAACATCTCAATACCTGTCTGCTTTTGATGGGTATTGGAAATTGGGGGTTGCAGGGCTTGTATAGCTTTGCTCTCCTTGAATGTATTAGTGGAGAAAAAGAAAGCTCATTGAACTCCATTGGTGTGATAAAATCTATCAATGCCAGGAACAATGTCCAAGTTTCTCTGCTGTCTAATCCATGTCCAGTTTGGACCTCATTTGACCAACT

>Amanita_fuliginea HKAS75782 (JX998022)

GGATCATTAAAGAAATGAACCTTGAGACTGTCGCTGGCCCCTCCTGCAGATGGAGTTGGCATGTGCACGTCTCTGGTCATTACCAATTCCACCTGTGCACAATTGTAGACACTTGGGAATGAGAGACATAGGCTGCTTGGCTGGTCTCTTGAGAGAGAGAAATGGAAAAAATCTGGGTGTCTATGCCATTTTTATTAAACACTAGTTGCATGTTTATAGAATGAATGATGATTTGATTGAATATGAAATACAACTTTCAACAACGGATCTCTTGGCTCTCGCATCGATGAAGAACGCAGCGAAATGCGATAAGTAATGTGAATTGCAGAATTCAGTGAATCATCGAATCTTTGAACGCACCTTGCGCTCCTTGGCATTCTGAGGAGCATGCCTGTTTGAGTGTCATTAACATCTCAATACCTGTCTGCTTTTGATGGGTATTGGAAATTGGGGGTTGCAGGGCTTGTATAGCTTTGCTCTCCTTGAATGTATTAGTGGAGAAAAAGAAAGCTCATTGAACTCCATTGGTGTGATAAAATCTATCAATGCCAGGAACAATGTCCAAGTTTCTCTGCTGTCTAATCCATGTCCAGTTTGGACCTCATTTGACCAACTTGACCTCAAATCAGGTAGGACTACCCGCTGA

>Amanita_molliuscula HKAS75555 (KJ466408)

GGATCATTAGTGAAATGAACCATGAGGCTGTTGCTGGCCCTTGTGGGCATGTGCACGTCTCTCTGGTCATTACCAAATTCCACCTGTGCACACTTTGTAGACACTTGGGAATGCAGWGACTTTGACCAGTCTCTTGAGGAGTTGAAATCTGGGTGTCTATGCCATTTTATCAAAACCTAGTTGCATGTCTATAGAATGACAATTTGATTTATATGTAAAAATACAACTTTCAACAATGGATCTCTTGGCTCTCGCATCGATGAAGAACGCAGCGAAATGCGATAAGTAATGTGAATTGCAGAATTCAGTGAATCATCGAATCTTTGAACGCACCTTGCGCTCCTTGGCATTCCAAGGAGCATGCCTGTTTGAGTGTCATTAAAAGTCTCAAGACCCTGTCAGCTTTTTGTTGATAGGTATTGGATTTTGGGGGTTTGCAGGCTTTTCTCAGATAAGGCCAGCTCTCCTTGAATATATTAGTGGAGATGAAAGCCATTTGAAACTCCATTGGTGTGATAAAAATCTATCAATGCCAGGAGCAATGTTGGTTCTCTCTGCTGTTTAATTGTCTGTGGAAATGGACGAATTGACCAACTTGACCTCAA

>Amanita_molliuscula HKAS77324 (KJ466409)

GGATCATTAGTGAAATGAACCATGAGGCTGTTGCTGGCCCTTGTGGGCATGTGCACGTCTCTCTGGTCATTACCAAATTCCACCTGTGCACACTTTGTAGACACTTGGGAATGCAGAGACTTTGACCAGTCTCTTGAGGAGTTGAAATCTGGGTGTCTATGCCATTTTATCAAAACCTAGTTGCATGTCTATAGAATGACAATTTGATTTATATGTAAAAATACAACTTTCAACAATGGATCTCTTGGCTCTCGCATCGATGAAGAACGCAGCGAAATGCGATAAGTAATGTGAATTGCAGAATTCAGTGAATCATCGAATCTTTGAACGCACCTTGCGCTCCTTGGCATTCCAAGGAGCATGCCTGTTTGAGTGTCATTAAAAGTCTCAAGACCCTGTCAGCTTTTTGTTGATAGGTATTGGATTTTGGGGGTTTGCAGGCTTTTCTCAGATAAGGCCAGCTCTCCTTGAATATATTAGTGGAGATGAAAGCCATTTGAAACTCCATTGGTGTGATAAAAATCTATCAATGCCAGGAGCAATGTTGGTTCTCTCTGCTGTTAAATTGTCTGTGGAAATGGGACGAATTGACCAACTTGACCTCAA

>Amanita_molliuscula Jilin-China

GGATCATTAGTGAAATGAACCATGAGGCTGTTGCTGGCCCTTGTGGGCATGTGCACGTCTCTCTGGTCATTACCAATTCCACCTGTGCACACTTTGTAGACACTTGGGAATGCAGAGACTTTGACCAGTCTCTTGAGGAGTTGAAATCTGGGTGTCTATGCCATTTTATCAAAACCTAGTTGCATGTCTATAGAATGACAATTTGATTTATATGTAAAAATACAACTTTCAACAATGGATCTCTTGGCTCTCGCATCGATGAAGAACGCAGCGAAATGCGATAAGTAATGTGAATTGCAGAATTCAGTGAATCATCGAATCTTTGAACGCACCTTGCGCTCCTTGGCATTCCAAGGAGCATGCCTGTTTGAGTGTCATTAAAAGTCTCAAGACCCTGTCAGCTTTTTGTTGATAGGTATTGGATTTTGGGGGTTTGCAGGCTTTTCTCAGATAAGGCCAGCTCTCCTTGAATATATTAGTGGAGATGAAAGCCATTTGAAACTCCATTGGTGTGATAAAAATCTATCAATGCCAGGAGCAATGTTGGTTCTCTCTGCTGTTTTAATTGTCTGTGGAAATGGAAATGGACAAATTGACCAACTTGACCTCAA

>Amanita_pallidorosea HKAS100625 (MH508484)

AAAAGTCGTAACAAGGTTTCCGTAGGTGAACCTGCGGAAGGATCATTACTGAAATGAACCTTGAGGCTGTTGCTGGCCCATCTGGGCATGTGCACGTCTCTGGTCATTACCAATTCCACCTGTGCACACTTGTAGACACTTGGGAATGAGAGACTTTGACCAGTCTCTTGAGAGAATTGAAATCTGGGTGTCTATGCCATTTTACCAAACACTAGTTGCATGTTTATAGAATGATTATTTGATTGAATATAAATACAACTTTCAACAACGGATCTCTTGGCTCTCGCATCGATGAAGAACGCAGCGAAATGCGATAAGTAATGTGAATTGCAGAATTCAGTGAATCATCGAATCTTTGAACGCACCTTGCGCTCCTTGGCATTCCGAGGAGCATGCCTGTTTGAGTGTCATTAACACCTCAAGACCTGTCTGCTTTTGATAGGTATTGGATTTTGGGAGTTGCAGGCTGTTTCAGATATAGCTTGCTCTCCTGGAATGTATTAGTGGAGAAAAGCTGTTGAACTCCATTGGTGTGATAAAATCTATCAATGCCAGGAGCAACATCAAGTGGTCTCTGCTGTCTAACCCTAACTGTCTGACCC

>Amanita_pallidorosea HKAS61937 (KJ466382)

GGATCATTACTGAAATGAACCTTGAGGCTGTCGCTGGCCCATCTGGGCATGTGCACGTCTCTGGTCATTACCAATTCCACCTGTGCACACTTGTAGACACTTGGGAATGAGAGACTTTGACCAGTCTCTTGAGAGAATTGAAATCTGGGTGTCTATGCCATTTTACCAAACACTAGTTGCATGTTTATAGAATGATTATTTGATTGAATATATAAATACAACTTTCAACAACGGATCTCTTGGCTCTCGCATCGATGAAGAACGCAGCGAAATGCGATAAGTAATGTGAATTGCAGAATTCAGTGAATCATCGAATCTTTGAACGCACCTTGCGCTCCTTGGCATTCCGAGGAGCATGCCTGTTTGAGTGTCATTAACACCTCAAGACCTGTCTGCTTTTGATAGGTATTGGATTTTGGGAGTTGCAGGCTGTTTCAGATATGCTTGCTCTCCTTGAATGTATTAGTGGAGAAAAGCTGTTGAACTCCATTGGTGTGATAAAATCTATCAATGCCAGGAGCAACATCAAGTGGTCTCTGCTGTCTAACCCTAACTGTCTGACTCACTTGACCTCAA

>Amanita_pallidorosea HKAS75786 (JX998037)

GGATCATTACTGAAATGAACCTTGAGGCTGTTGCTGGCCCATCTGGGCATGTGCACGTCTCTGGTCATTACCAATTCCACCTGTGCACACTTGTAGACACTTGGGAATGAGAGACTTTGACCAGTCTCTTGAGAGAATTGAAATCTGGGTGTCTATGCCATTTTACCAAACACTAGTTGCATGTTTATAGAATGATTATTTGATTGAATATAAATACAACTTTCAACAACGGATCTCTTGGCTCTCGCATCGATGAAGAACGCAGCGAAATGCGATAAGTAATGTGAATTGCAGAATTCAGTGAATCATCGAATCTTTGAACGCACCTTGCGCTCCTTGGCATTCCGAGGAGCATGCCTGTTTGAGTGTCATTAACACCTCAAGACCTGTCTGCTTTTGATAGGTATTGGATTTTGGGAGTTGCAGGCTGTTTCAGATATAGCTTGCTCTCCTGGAATGTATTAGTGGAGAAAAGCTGTTGAACTCCATTGGTGTGATAAAATCTATCAATGCCAGGAGCAACATCAAGTGGTCTCTGCTGTCTAACCCTAACTGTCTGACCCACTTGACCTCAAATCAGGTAGGACTACCCGCTGA

>Amanita_rimosa HKAS75777 (JX998018)

CATTAAAGAGATGAACCTTGAGGCTGTTGCTGGCTCATATATAGGCATGTGCACGCCTCTGGTCATTATTAATTCCACCTGTGCATGCTTGTAGACACTTGGGAATGAGAGACTTTAACCAGCCCCTTGAGAAATTGAACTTTCCAATCTCTGGGTGTCTATGCCATTTTTAAAACACCAGTTGCATGTTTATAGAATGATATGATTACCATGTAAAATACAACTTTCAACAACGGATCTCTTGGCTCTCGCATCGATGAAGAACGCAGCGAAATGCGATAAGTAATGTGAATTGCAGAATTCAGTGAATCATCAAATCTTTGAACGCACCTTGCGCTCCTTGGCATTCTGAGGAGCATGCCTGTTTGAGTGTCATTAATATCTCAAGACCTGTCTGCTTTTGATAGGTTTCGGATTTATGGGGGTTGCAGGCTGTTTCAAATAGCTTGCTCTCCTTGAATGTATTATTAGTGGAGGGAAGAAAAAGTCATTAAACTCCATTGGTGTGATAAAATGGTATCAATGCCAGGAGSAATGAACAACAGTTCTCTCAGCTAACTGTCTGTAGTAAAACATGGACAACTTGACCAACTTGACCTCAAATCAGGTAGGACTACCCGCTGA

>Amanita_rimosa HKAS75779 (JX998020)

GGATCATTAAAGAAAATGAACCTTGAGGCTGTTGCTGGCTCATATATAGGCATGTGCACGCCTCTGGTCATTATTAATTCCACCTGTGCATGCTTGTAGACACTTGGGAATGAGAGACTTTAACCAGCCCCTTGAGAAATTGAACTTTCCAATCTCTGGGTGTCTATGCCATTTTTAAAACACCAGTTGCATGTTTATAGAATGATATGATTACCATGTAAAATACAACTTTCAACAACGGATCTCTTGGCTCTCGCATCGATGAAGAACGCAGCGAAATGCGATAAGTAATGTGAATTGCAGAATTCAGTGAATCATCAAATCTTTGAACGCACCTTGCGCTCCTTGGCATTCTGAGGAGCATGCCTGTTTGAGTGTCATTAATATCTCAAGACCTGTCTGCTTTTGATAGGTTTCGGATTTATGGGGGTTGCAGGTTGTTTCAAATAGCTTGCTCTCCTTGAATGTATTATTAGTGGAGGGAAGAAAAAGTCATTAAACTCCATTGGTGTGATAAAATGGTATCAATGCCAGGAGCAATGAACAACAGTTCTCTCAGCTAACTGTCTGTAGTAAAACAATGGACAACTTGACCAACTTGACCTCAAATCAGGTAGGACTACCCGCTGA

>Amanita_rimosa HKAS77105 (KJ466391)

GGATCATTAAAGAAAATGAACCTTGAGGCTGTTGCTGGCTCATATATAGGCATGTGCACGCCTCTGGTCATTATTAATTCCACCTGTGCATGCTTGTAGACACTTGGGAATGAGAGACTTTAACCAGCCCCTTGAGAAATTGAACTTTCCAATCTCTGGGTGTCTATGCCATTTTTAAAACACCAGTTGCATGTTTATAGAATGATATGATTACCATGTAAAATACAACTTTCAACAACGGATCTCTTGGCTCTCGCATCGATGAAGAACGCAGCGAAATGCGATAAGTAATGTGAATTGCAGAATTCAGTGAATCATCAAATCTTTGAACGCACCTTGCGCTCCTTGGCATTCTGAGGAGCATGCCTGTCTGAGTGTCATTAATATCTCAAGACCTGTCTGCTTTTGATAGGTTTCGGATTTATGGGGGTTGCAGGTTGTTTCAAATAGCTTGCTCTCCTTGAATGTATTATTAGTGGAGGGAAGAAAAAGTCATTAAACTCCATTGGTGTGATAAAATGGTATCAATGCCAGGAGCAATGAACAACAGTTCTCTCAGCTAACTGTCTGTAGTAAAACAATGGACAACTTGACCAACTTGACCTCAA

>Amanita_subjunquillea HKAS100581 (MH508622)

AAAGTCGTAACAAGGTTTCCGTAGGTGAACCCGCGGAAGGATCATTAATGAAATGAACCTTGAGGCTGTCGCTGGCCCCTCTGGGGCATGTGCACGTCTCTGGTCATTACTAATTCCACCTGTGCACACTTGTAGACACTTGGGAATGAGAGACCTTGACCAGTCTCTTGAGAAATTGAACATCTGGGTGTCTATGCCATTTTATTAAACACTAGTTGCATGTTTATAGAATGATGATTTGATTAAATATAAAGTACAACTTTCAACAACGGATCTCTTGGCTCTCGCATCGATGAAGAACGCAGCGAAATGCGATAAGTAATGTGAATTGCAGAATTCAGTGAATCATCGAATCTTTGAACGCACCTTGCGCTCCTTGGCATTCCGAGGAGCATGCCTGTTTGAGTGTCATTAAATTCTCAAGACCTGTCTGCTTTTTTGATAGGTATTGGATTTTGGGGGTTGCAGGCTGTTTCAAATAAATAGCCTTGCTCTCCTTGAATGTATTAGTGGAGAAAAGCCATTGAACTCCATTGGTGTGATAAAACCTATCAATGCCAGGAGCAATATCACTACTTCTCTCTGCTGTCTAACTGTGACTGTCTGTATTAATTAGTATGGATGGGGACAACTTGACCAACTGACCT

>Amanita_subjunquillea HKAS54509 (KJ466422)

GGATCATTAATGAAATGAACCTTGAGGCTGTCGCTGGCCCCTCTGGGGCATGTGCACGTCTCTGGTCATTACTAATTCCACCTGTGCACACTTGTAGACACTTGGGAATGAGAGACCTTGACCAGTCTCTTGAGAAATTGAACATCTGGGTGTCTATGCCATTTTATTAAACACTAGTTGCATGTTTATAGAATGATGATTTGATTAAATATAAAGTACAACTTTCAACAACGGATCTCTTGGCTCTCGCATCGATGAAGAACGCAGCGAAATGCGATAAGTAATGTGAATTGCAGAATTCAGTGAATCATCGAATCTTTGAACGCACCTTGCGCTCCTTGGCATTCCGAGGAGCATGCCTGTTTGAGTGTCATTAAATTCTCAAGACCTGTCTGCTTTTTTGATAGGTATTGGATTTTGGGGGTTGCAGGCTGTTTCAAATAAATAGCCTTGCTCTCCTTGAATGTATTAGTGGAGAAAAGCCATTGAACTCCATTGGTGTGATAAAACCTATCAATGCCAGGAGCAATATCACTACTTCTCTCTGCTGTCTAACTGTGACTGTCTGTATTAATTAGTATGGATGGGGACAACTTGACCAACTGACCTCAA

>Amanita_subjunquillea HKAS77345 (KJ466426)

GGATCATTAATGAAATGAACCTTGAGGCTGTCGCTGGCCCCTCTGGGGCATGTGCACGTCTCTGGTCATTACTAATTCCACCTGTGCACACTTGTAGACACTTGGGAATGAGAGACCTTGACCAGTCTCTTGAGAAATTGAACATCTGGGTGTCTATGCCATTTTATTAAACACTAGTTGCATGTTTATAGAATGATGATTTGATTAAATATAAAGTACAACTTTCAACAACGGATCTCTTGGCTCTCGCATCGATGAAGAACGCAGCGAAATGCGATAAGTAATGTGAATTGCAGAATTCAGTGAATCATCGAATCTTTGAACGCACCTTGCGCTCCTTGGCATTCCGAGGAGCATGCCTGTTTGAGTGTCATTAAATTCTCAAGACCTGTCTGCTTTTTTGATAGGTATTGGATTTTGGGGGTTGCAGGCTGTTTCAAATAAATAGCCTTGCTCTCCTTGAATGTATTAGTGGAGAAAAGCCATTGAACTCCATTGGTGTGATAAAACCTATCAATGCCAGGAGCAATATCACTACTTCTCTCTGCTGTCTAACTGTGACTGTCTGTATTAATTAGTATGGATGGGGACAACTTGACCAACTTGACCTCAA

>Amanita_virosa HKAS56694 (JX998030)

GGATCATTACTGAAATGAACCTTGAGGCTGTTGCTGGCCCATCTGGGCATGTGCACGTCTCTGGTCATTACCAATTCCACCTGTGCACACACTTGTAGACACTTGGGAATGAGAGACTTTGACCAGTCTCTTGAGAGATTTCATATCTGGGTGTCTATGCCTTTTTATTACACACTAGTTGCATGTTTATAGAATGATGATTTGATTAAATATAAAATACAACTTTCAACAACGGATCTCTTGGCTCTCGCATCGATGAAGAACGCAGCGAAATGCGATAAGTAATGTGAATTGCAGAATTCAGTGAATCATCGAATCTTTGAACGCACCTTGCACTCCTTGGCATTCCGAGGAGCATGCCTGTTTGAGTGTCATTAACATCTCAAGACCTGTCTGTTTTTGATAGGTATTGGATTTTTGGGGGTTTGCAGGCTGTTTCAGATAGCTTGCTCTCCTTGAATGTATTAGTGGAGAAAGAGCCATTGAACTCCATTGGTGTGATAAAATCTATCAATGCCAGGAGCCATGTTAGTTCTCTCTGCTGTCTAACCGCTAACAGTTGTCTGTAAAAAAAATGGACAACTTGACCAACTTGACCTCAAATCAGGTAGGACTACCCGCTGA

>Amanita_virosa HKAS84859 (KR862367)

TAAAAGTCGTAACAAGGTTTCCGTAGGTGAACCTGCGGAAGGATCATTACTGAAATGAACCTTGAGGCTGTTGCTGGCCCATCTGGGCATGTGCACGTCTCTGGTCATTACCAATTCCACCTGTGCACACACTTGTAGACACTTGGGAATGAGAGACTTTGACCAGTCTCTTGAGAGATTTCATATCTGGGTGTCTATGCCTTTTTATTACACACTAGTTGCATGTTTATAGAATGATGATTTGATTAAATATAAAATACAACTTTCAACAACGGATCTCTTGGCTCTCGCATCGATGAAGAACGCAGCGAAATGCGATAAGTAATGTGAATTGCAGAATTCAGTGAATCATCGAATCTTTGAACGCACCTTGCACTCCTTGGCATTCCGAGGAGCATGCCTGTTTGAGTGTCATTAACATCTCAAGACCTGTCTGTTTTTGATAGGTATTGGATTTTTGGGGGTTTGCAGGCTGTTTCAGATAGCTTGCTCTCCTTGAATGTATTAGTGGAGAAAGAGCCATTGAACTCCATTGGTGTGATAAAATCTATCAATGCCAGGAGCCATGTTAGTTCTCTCTGCTGTCTAACCGCTAACAGTTGTCTGTAAAAAAAATGGACAACTTGACCAACTGACCTCAAAT

>Amanita_virosa HKAS90176 (MH508650)

GGATCATTACTGAAATGAACCTTGAGGCTGTTGCTGGCCCATCTGGGCATGTGCACGTCTCTGGTCATTACCAATTCCACCTGTGCACACACTTGTAGACACTTGGGAATGAGAGACTTTGACCAGTCTCTTGAGAGATTTCATATCTGGGTGTCTATGCCTTTTTATTACACACTAGTTGCATGTTTATAGAATGATGATTTGATTAAATATAAAATACAACTTTCAACAACGGATCTCTTGGCTCTCGCATCGATGAAGAACGCAGCGAAATGCGATAAGTAATGTGAATTGCAGAATTCAGTGAATCATCGAATCTTTGAACGCACCTTGCACTCCTTGGCATTCCGAGGAGCATGCCTGTTTGAGTGTCATTAACATCTCAAGACCTGTCTGTTTTTGATAGGTATTGGATTTTTTGGGGGTTTGCAGGCTGTTTCAGATAGCTTGCTCTCCTTGAATGTATTAGTGGAGAAAGAGCCATTGAACTCCATTGGTGTGATAAAATCTATCAATGCCAGGAGCCATGTTAGTTCTCTCTGCTGTCTAACCACTAACAGTTGTCTGTAAAAAAAATGGACAACTTGACCAACTTGACCTCAA

>Amanita_bisporigera RET505-7 (KP224347)

AGGATCATTACTGAAATGAACCTTGAGGCTGTCGCTGGCCCATCTGGGCATGTGCACGTCTCTGGTCATTACCAATTCCACCTGTGCACACTTGTAGACACTTGGGAATGAGAGACTTTGACCGGTCTCTTGAGGGAATTGAACTCTGGGTGTCTATGCCATTTTATCAAACACTAGTTGCATGTTTATAGAATGACGATTTGATTGAATATAAAATACAACTTTCAACAACGGATCTCTTGGCTCTCGCATCGATGAAGAACGCAGCGAAATGCGATAAGTAATGTGAATTGCAGAATTCAGTGAATCATCGAATCTTTGAACGCACCTTGCGCTCCTTGGCATTCCGAGGAGCATGCCTGTTTGAGTGTCATTAACATCTCAAGACCTGTCTGCTTTTGATAGGTATTGGATTTTGGGAGTTGCAGGCTGTTTCAGATATAGCTTGCTCTCCTTGAATGTATTAGTGGAGAAAAGCTGTTGAACTCCATTGGTGTGATAAAATCTATCAATGCCAGGAGCAACGTCGAGTTGTCTCTGCTGTCTAACCCTAACTGTCTGACCCACTTGACCTCAAATCAGGTAGGACTACCCGCTGAACTTAAGCATATCAATAAGC

>Amanita_bisporigera RET377-9 (KJ466374)

GGATCATTACTGAAATGAACCTCGAGGCTGTCGCTGGCCCATCTGGGCATGTGCACGTCTCTGGTCATTACCAATTCCACCTGTGCACACTTGTAGACACTTGGGAATGAGAGACTTTGACCGGTCTCTCGAGGGAATTGAACTCTGGGTGTCTATGCCATTTTATCAAACACTAGTTGCATGTTTATAGAATGACGATTTGATTGAATATAAAATACAACTTTCAACAACGAATCTCTTGGCTCTCGCATCGATGAAGAACGCAGCGAAATGCGATAAGTAATGTGAATTGCAGAATTCAGTGAATCATCGAATCTTTGAACGCACCTTGCGCTCCTTGGCATTCCGAGGAGCATGCCTGTTTGAGTGTCATTAACATCTCAAGACCTGTCTGCTTTTGATAGGTATTGGATTTTGGGAGTTGCAGGCTGTTCCAGATATAGCTTGCTCTCCTTGAATGTATTAGTGGAGAAAAGCTGTTGAACTCCATTGGTGTGATAAAATCTATCAATGCCAGGAGCAACGTCGAGTTGTCTCTGCTGTCTAACCCTAACTGTCTGACCCACTTGACCTCAA

>Amanita_bisporigera RET643-5 (KR919764)

AGGATCATTACTGAAATGAACCTCGAGGCTGTCGCTGGCCCATCTGGGCATGTGCACGTCTCTGGTCATTACCAATTCCACCTGTGCACACTTGTAGACACTTGGGAATGAGAGACTTTGACCGGTCTCTTGAGGGAATTGAACTCTGGGTGTCTATGCCATTTTATCAAACACTAGTTGCATGTTTATAGAATGACGATTTGATTGAATATAAAATACAACTTTCAACAACGGATCTCTTGGCTCTCGCATCGATGAAGAACGCAGCGAAATGCGATAAGTAATGTGAATTGCAGAATTCAGTGAATCATCGAATCTTTGAACGCACCTTGCGCTCCTTGGCATTCCGAGGAGCATGCCTGTTTGAGTGTCATTAACATCTCAAGACCTGTCTGCTTTTGATAGGTATTGGATTTTGGGAGTTGCAGGCTGTTTCAGATATAGCTTGCTCTCCTTGAATGTATTAGTGGAGAAAAGCTGTTGAACTCCATTGGTGTGATAAAATCTATCAATGCCAGGAGCAACGTCGAGTTGTCTCTGCTGTCTAACCCTAACTGTCTGACCCACTTGACCTCAAATCAGGTAGGACTACCCGCTGAACTTAAGCATATCAATAAGCGGAGGAAAAGAAACTAACAAGGATTCCCCTAGTAACTGCGAGTGAAGCGGGAAAAGCTCAAATTTAAAATCTGGCAGATTTTTGTCTGTCCGAGT

>Amanita_phalloides RET118-6 (GQ221841)

AAGGATCATTAATGAAATGAACCTTGAGGCTGTCGCTGGCCCCTCTGGGGCATGTGCACGTCTCTGGTCATTACCAATTCCACCTGTGCACACTTGTAGACACTTGGGAATGAGAGACCTTGACCAGTCTCTTGAGAAGTTGAAAATCTGGGTGTCTATGCCATTTTATTAAACACTAGTTGCATGTTTATAGAATGATGATTTGATTAAATATAAAGTACAACTTTCAACAACGGATCTCTTGGCTCTCGCATCGATGAAGAACGCAGCGAAATGCGATAAGTAATGTGAATTGCAGAATTCAGTGAATCATCGAATCTTTGAACGCACCTTGCGCTCCTTGGCATTCCGAGGAGCATGCCTGTTTGAGTGTCATTAAATTCTCAAGACCTGTCTGCTTTTTTGATAGGTATTGGATTTTTGGGGGTTGCAGGCTGTTTCAAATAAAAATAGCCTTGCTCTCTTTGAATGTATTAGTGGAGAAAAGCCATTGAACTCCATTGGTGTGATAAAACCTATCAATGCCAGGAGCAATATCACTTCTCTCTGCTGTCTAACTGTGACTGTCTGTATAAATTTATATGGATGGGGACAACTTGACCAACTTGACCTCAAATCAGGTAGGACTACCCGCTGAACTTAA

>Amanita_phalloides GDGM40312 (KC755034)

TCCGTAGGTGAACCTGCGGAAGGATCATTAATGAAATGAACCTTGAGGCTGTCGCTGGCCCCTCTGGGGCATGTGCACGTCTCTGGTCATTACCAATTCCACCTGTGCACACTTGTAGACACTTGGGAATGAGAGACCTTGACCAGTCTCTTGAGAAGTTGAAAATCTGGGTGTCTATGCCATTTTATTAAACACTAGTTGCATGTTTATAGAATGATGATTTGATTAAATATAAAGTACAACTTTCAACAACGGATCTCTTGGCTCTCGCATCGATGAAGAACGCAGCGAAATGCGATAAGTAATGTGAATTGCAGAATTCAGTGAATCATCGAATCTTTGAACGCACCTTGCGCTCCTTGGCATTCCGAGGAGCATGCCTGTTTGAGTGTCATTAAATTCTCAAGACCTGTCTGCTTTTTTGATAGGTATTGGATTTTTGGGGGTTGCAGGCTGTTTCAAATAAAAATAGCCTTGCTCTCTTTGAATGTATTAGTGGAGAAAAGCCATTGAACTCCATTGGTGTGATAAAACCTATCAATGCCAGGAGCAATATCACTTCTCTCTGCTGTCTAACTGTGACTGTCTGTATAAATTTATATGGATGGGGACAACTTGACCAACTTGACCTCAAATCAGGTAGGACTACCCGCTGAACTTAAGCATATCAATAA

>Amanita_phalloides RET053-2 (KF561975)

GGAAGTAAAAGTCGTAACAAGGTTTCCGTAGGTGAACCTGCGGAAGGATCATTAATGAAATGAACCTTGAGGCTGTCGCTGGCCCCTCTGGGGCATGTGCACGTCTCTGGTCATTACCAATTCCACCTGTGCACACTTGTAGACACTTGGGAATGAGAGACCTTGACCAGTCTCTTGAGAAGTTGAAAATCTGGGTGTCTATGCCATTTTATTAAACACTAGTTGCATGTTTATAGAATGATGATTTGATTAAATATAAAGTACAACTTTCAACAACGGATCTCTTGGCTCTCGCATCGATGAAGAACGCAGCGAAATGCGATAAGTAATGTGAATTGCAGAATTCAGTGAATCATCGAATCTTTGAACGCACCTTGCGCTCCTTGGCATTCCGAGGAGCATGCCTGTTTGAGTGTCATTAAATTCTCAAGACCTGTCTGCTTTTTTGATAGGTATTGGATTTTTGGGGGTTGCAGGCTGTTTCAAATAAAAATAGCCTTGCTCTCTTTGAATGTATTAGTGGAGAAAAGCCATTGAACTCCATTGGTGTGATAAAACCTATCAATGCCAGGAGCAATATCACTTCTCTCTGCTGTCTAACTGTGACTGTCTGTATAAATTTATATGGATGGGGACAACTTGACCAACTTGACCTCAAATCAGGTAGGACTACCCGCT

>Amanita_subpallidorosea LHJ140923-41 (KP691683)

TCCGTAGGTGAACCTGCGGAAGGATCATTACTGAAATGAACCTTGAGGCTGTCGCTGGCCCATCTGGGCATGTGCACGTCTCTGGTCATTACCAATTCCACCTGTGCACACTTGTAGACACTTGGGAATGAGAGACTTTGACCAGTCTCTTGAGAGGATTGAAATCTGAGTGTCTATGCCATTTTATTACACACTAGTTGCATGTTTATAGAATGATAATTTGATTAAATGTAAAATACAACTTTCAACAACGGATCTCTTGGCTCTCGCATCGATGAAGAACGCAGCGAAATGCGATAAGTAATGTGAATTGCAGAATTCAGTGAATCATCGAATCTTTGAACGCACCTTGCGCTCCTTGGCATTCCGAGGAGCATGCCTGTTTGAGTGTCATTAACATCTCAAGACCTGTCTGCTTTTGATAGGTGTTGGATTTTTGGGTGTTGCAGGCTTTTTCAGATAGCTTGCTCTCCTTGAATGTATTAGTGGAGAAGAGCCATTGAACTCCATTGGTGTGATAAAATCTATCAATGCCAGGAGCCATGTTAGTTCTCTCTGCTGTCTAACTCTCACAGTTGTCTGTAAAAAATGGACAACTTGACCAACTGACCTCAAATCAG

>Amanita_subpallidorosea HKAS77350 (KJ466400)

GGATCATTACTGAAATGAACCTTGAGGCTGTCGCTGGCCCATCTGGGCATGTGCACGTCTCTGGTCATTACCAATTCCACCTGTGCACACTTGTAGACACTTGGGAATGAGAGACTTTGACCAGTCTCTTGAGAGGATTGAAATCTGAGTGTCTATGCCATTTTATTACACACTAGTTGCATGTTTATAGAATGATAATTTGATTAAATGTAAAATACAACTTTCAACAACGGATCTCTTGGCTCTCGCATCGATGAAGAACGCAGCGAAATGCGATAAGTAATGTGAATTGCAGAATTCAGTGAATCATCGAATCTTTGAACGCACCTTGCGCTCCTTGGCATTCCGAGGAGCATGCCTGTTTGAGTGTCATTAACATCTCAAGACCTGTCTGCTTTTGATAGGTGTTGGATTTTTGGGTGTTGCAGGCTGTTTCAGAAAGCTTGCTCTCCTTGAATGTATTAGTGGAGAAGAGCCATTGAACTCCATTGGTGTGATAAAATCTATCAATGCCAGGAGCCATGTTAGTTCTCTCTGCTGTCTAACTCTCACAGTTGTCTGTAAAAAATGGACAACTTGACCAACTG

>Amanita_subpallidorosea LHJ140923-41 (KP691683)

TCCGTAGGTGAACCTGCGGAAGGATCATTACTGAAATGAACCTTGAGGCTGTCGCTGGCCCATCTGGGCATGTGCACGTCTCTGGTCATTACCAATTCCACCTGTGCACACTTGTAGACACTTGGGAATGAGAGACTTTGACCAGTCTCTTGAGAGGATTGAAATCTGAGTGTCTATGCCATTTTATTACACACTAGTTGCATGTTTATAGAATGATAATTTGATTAAATGTAAAATACAACTTTCAACAACGGATCTCTTGGCTCTCGCATCGATGAAGAACGCAGCGAAATGCGATAAGTAATGTGAATTGCAGAATTCAGTGAATCATCGAATCTTTGAACGCACCTTGCGCTCCTTGGCATTCCGAGGAGCATGCCTGTTTGAGTGTCATTAACATCTCAAGACCTGTCTGCTTTTGATAGGTGTTGGATTTTTGGGTGTTGCAGGCTTTTTCAGATAGCTTGCTCTCCTTGAATGTATTAGTGGAGAAGAGCCATTGAACTCCATTGGTGTGATAAAATCTATCAATGCCAGGAGCCATGTTAGTTCTCTCTGCTGTCTAACTCTCACAGTTGTCTGTAAAAAATGGACAACTTGACCAACTGACCTCAAATCAG
