# Supplementary material for: Differential Expression of Amanitin Biosynthetic Genes and Novel Cyclic Peptides in Amanita molliuscula
Source: J Fungi (Basel). 2021 May 14;7(5):384. doi: 10.3390/jof7050384 (PMC8156247; doi:10.3390/jof7050384)
Supplement: Supplementary file 1 [file jof-07-00384-s001.zip › supplementary files/Supplementary file 2.docx]

| Genes | Primers |
| --- | --- |
| *AmAMA1* | 5’-GGCTAACCAAAACCACAAGACC-3’ (forward) and 5’-GACGTATAAAAGGGATTGTTGACC-3’ (reverse) |
| *AmAMA2-1* | 5’-GGCCGATATGATTGATTTCTGATT-3’ (forward) and 5’-CTGTGGATCGTTCGGTCATACTAA-3’ (reverse) |
| *AmAMA2-2* | 5’-ACTGGGGCATCGTGAGCA-3’ (forward) and 5’-CGTATAAAAGGAATCGTGAACC-3’ (reverse) |
| *AmAMA3* | 5’-TTCAGCACCCAACTCCCATTC-3’ (forward) and 5’-TGCTATATGGTTCGATGGACTTTG-3’ (reverse) |
| *AmCylK1* | 5’-CGCTCAGCTTCTACATACCACTCT-3’ (forward) and 5’-GTTCGCTTGTCACGTCACTCTTAT-3’ (reverse) |
| *AmCylK2* | 5’-CATGTGTTGACTCGCTTCTACTTC-3’ (forward) and 5’-GACGAAAGAACGACAAAGACAAAC-3’ (reverse) |
| *AmPOPB* | 5’-CATCCACCCGTACAGACATCAGAC-3’ (forward) and 5’-CGGCATTTCATTGACGGCATAGT-3’ (reverse) |

Supplementary file 2. The primers for amanitin biosynthetic genes.
