# Supplementary material for: Differential Expression of Amanitin Biosynthetic Genes and Novel Cyclic Peptides in Amanita molliuscula
Source: J Fungi (Basel). 2021 May 14;7(5):384. doi: 10.3390/jof7050384 (PMC8156247; doi:10.3390/jof7050384)
Supplement: Supplementary file 1 [file jof-07-00384-s001.zip › supplementary files/Supplementary file 3.docx]

|  | **Peptides and proteins** | pvalue | Am1 | Am2 | Am3 |
| --- | --- | --- | --- | --- | --- |
| **Toxin genes** | MSDINATRLP IWGIGCNP CVGDDVTTLLTRGEALC | 0.961527995 | 15.83513075 | 16.38853318 | 13.81035301 |
|  | MSDINATRLA IWGIGCNP CVGDDVTALLTRGEALC | 0.95638691 | 8.907261047 | 9.218549916 | 9.864537866 |
|  | MSDINATRLA IWGIGCNP CVGDDVTALLTRGEALC | 0.984006548 | 26.72178314 | 26.63136642 | 27.62070603 |
|  | MSDINATRLA IWGIGCDP CVGDDVTALLTRGEALC | 0.986708726 | 16.82482642 | 17.41281651 | 16.76971437 |
|  | MSNINALRLP GFGFIP YASGDVDYTLTRGESLS | 0.834122837 | 86651.81486 | 87606.9527 | 86497.20028 |
|  | MSDINATRFP GKVNPP YVGDDVDDIIIRGEKLC | 0.902688423 | 823.426799 | 852.2037256 | 766.4745922 |
|  | MSNINASRLP IWAAFFRFP CVGDEVDGILRSGESLC | 0.963132604 | 24.7423918 | 26.63136642 | 24.66134467 |
|  | MTDINATRLP FFWILIPP CVDDVDNTVHSGDNLC | 0.992166417 | 49.48478359 | 50.18988288 | 49.32268933 |
|  | MSDINATRFP GKVFPP YVGDDVDDIIIRGEK | 0.963660418 | 17.81452209 | 16.38853318 | 17.75616816 |
|  | MSNINATRFP GKLFPP YVGDDVDDIIIRGDKLC | 0.992604488 | 4.948478359 | 5.12141662 | 4.932268933 |
|  | MSDINASRLP RLLVPRYP CIDEDAEAILRSGECL | 0.978336977 | 45.52600091 | 47.1170329 | 45.37687418 |
|  | MTDINATRLP ILFGFFLLP CVDGVDNTLHSGENLC | 0.937457504 | 8.907261047 | 7.169983268 | 8.87808408 |
|  | MSDINSIHLP GGYYQNT FVGDDVEGILNRGERLC | 0.985487586 | 70.2683927 | 71.69983268 | 70.03821885 |
|  | MTDINATRLP LNILPFHLPP CVDDVDNTLHSGENLC | 0.976445052 | 9.896956719 | 9.218549916 | 9.864537866 |
|  | MTDINATRLP FFIIFFIPP CVDDVDNTLHSGENLC | 0.987492902 | 14.84543508 | 15.36424986 | 14.7968068 |
|  | MTDINATRLP FFIIFFIPP CVDDVDNTLHSGENLC | 0.985613213 | 19.79391344 | 20.48566648 | 19.72907573 |
|  | MTDINATRLP WIFFFYPP CVDDVDNTLHSGENLC | 0.994181178 | 2.969087016 | 3.072849972 | 2.95936136 |
|  | MTDINATRLP LIFLPPFIPP CVDDVDNTLHSGENLC | 0.996519942 | 0.989695672 | 1.024283324 | 0.986453787 |
|  | MTDINATRLP LIFLPPFIPP CVDDVDNTLHSGENLC | 0.976206227 | 65.31991434 | 67.60269939 | 61.16013477 |
|  | MTDINATRLP WFFFFYPP CVDDVDNTLHSGENLC | 0.991938887 | 5.938174031 | 6.145699944 | 5.91872272 |
|  | MTDINATRLP FNILPLLLPP CVDDVDNTLHSGENLC | 0.992604488 | 4.948478359 | 5.12141662 | 4.932268933 |
|  | *POPB* | 0.993595101 | 12156.43194 | 12168.48589 | 12152.1242 |
| **Housekeeping genes** | *POPA* | 0.983647835 | 505.7344883 | 509.068812 | 506.0507925 |
|  | *rbp2* | 0.993595101 | 12156.43194 | 12168.48589 | 12152.1242 |

Supplementary file 3. Comparison of expression of amanitin biosynthetic genes among three technical replicates (Am1, Am2 and Am3).
