# Supplementary material for: Differential Expression of Amanitin Biosynthetic Genes and Novel Cyclic Peptides in Amanita molliuscula
Source: J Fungi (Basel). 2021 May 14;7(5):384. doi: 10.3390/jof7050384 (PMC8156247; doi:10.3390/jof7050384)
Supplement: Supplementary file 1 [file jof-07-00384-s001.zip › supplementary files/Supplementary file 4.docx]

Supplementary file 4. Genomic DNA and amino acid sequences of amanitin biosynthetic genes (introns underlined, start and stop codons in red, and core peptides in bold).

1. *AmAMA1*

Nucleotide sequence:

CCTCTTAAAGCTCCCAAATCACAATGTCTGACATCAATGCCACCCGTCTTCCCATCTGGGGAATCGGCTGTAACCCCTGCGTCGGTGACGACGTCACTACACTCCTCACCCGGGGCGAGGCGTAAGCAGAGTCTCTCTACAATAATGTACCAATGCACTTATGCCTTACATGTTAGCCTTTGCTAAATACCGCTCCAATGGTCCGCTGCTATGACTGGAAGGTATTGATCGCTTCATAACGACGAAACAAGGCAGTTGCACTGACTTAGTAGTGGACGAACAAGTTGTCGACGTTATCAGGCTTGGATCGTTGAGTCTGCGTCGGAAGTAGGACCTTTCCGTTTTGTGGCAAAACACGAGGCTAAATTGTCTTTTCTCAGACAACTCACATTCTCCTTTTTTCTGTTGGCGGATCCGTTGTCTCATTTGTAAAAATATAAAACCCACGTCGATGATCTGTGTTGTAGTCAGAATAAAGTTGTACTTTTGCCATGC

Amino acid sequence:

MSDINATRLP**IWGIGCNP**CVGDDVTTLLTRGEALC

2. *AmAMA2-1*

Nucleotide sequence:

CCTCTAAAGCTCCCAAACCACAATGTCTGACATCAATGCCACCCGTCTTGCCATCTGGGGAATCGGCTGTAACCCGTGCGTCGGTGACGATGTCACTGCACTCCTCACCCGCGGCGAGGCGTAAGCAGAATTTCTCTTAAATAATGTACCAATGCACTTATGCGTCGCGTATCAGTCTTTGCTAAATACGCAATCCATTGACCCGCTGCTATGACACGGAGGTATTATCATCTCACTTATTAACGATGATACAAGGCAGAAGTTGATTCAAACGTAGTAGTGGGCGATACAAGTTGTTGATCTTATCAGGCTTGGACCGTTGAGCCTGCGTCGGAAGTAGGCCCTTCTTGTTTCTGGAAAAACACAAGGCTAAATCGTCTTTTCTCAGACAACTTTCATTTCATTTTTTGGCGGACCCGTTGTCTCATTTATAAAAATATAAAACCCACGTCGATGACCTGTGTTATAGTCAATATGAAGTTGTACTGTGTTTCTTGT

Amino acid sequence:

MSDINATRLA**IWGIGCNP**CVGDDVTALLTRGEALC

3. *AmAMA2-2*

Nucleotide sequence:

CCTCTAAAGCTCCCAAACCACAATGTCTGACATCAATGCCACCCGTCTTGCCATCTGGGGAATCGGCTGTAACCCGTGCGTCGGTGACGATGTCACTGCACTCCTCACCCGCGGCGAGGCGTAAGCAGAATTTCTCTTAAATAATGTACCAATGCACTTATGCGTCGCGTATCAGTCTTTGCTAAATACGCAATCCATTGACCCGCTGCTATGACACGGAGGTATTATCATCTCACTTATTAACGATGATACAAGGCAGAAGTTGATTCAAACGTAGTAGTGGGCGATACAAGTTGTTGATCTTATCAGGCTTGGACCGTTGAGCCTGCGTCGGAAGTAGGCCCTTCTTGTTTCTGGAAAAACACAAGGCTAAATCGTCTTTTCTCAGACAACTTTCATTTCATTTTTTGGCGGACCCGTTGTCTCATTTTATAAAAATATAAAACCCACGTCGACGATCTGTGTTATAGTCAATATGAAGTTGTACTGTGTTTCTTGT

Amino acid sequence:

MSDINATRLA**IWGIGCNP**CVGDDVTALLTRGEALC

4. *AmAMA3*

Nucleotide sequence:

TGAACCTCTACGTTCAGCACCCAACTCCCATTCGACCACCTCTAAAGCTCCCAAACCACAATGTCTGACATCAATGCCACCCGTCTTGCCATCTGGGGAATCGGCTGTGACCCGTGCGTCGGTGACGATGTCACTGCACTCCTCACCCGCGGCGAGGCGTAAGCAGAATTTCTCTCCAATAATGTACCAATGCACTTATGCGTCGCGTATTAGTCTTTGCTAAATACGCAATCCATTGACCCGCTGCTATGACAAGGAGGTATTATCATCTCACTTATTAACGATGATACAAGGCAGAAGTTGATTCAAACGTAGTAGTGGGCGATACAAGTTGTTGATCTTATCAGGCTTGGACCGTTAAGCCTGCGTCGGAAGTAGGCCCTTCTTGTTTCTGGAAAAACACAAGGCTAAATCGTCTTTTCTCAGACAACTTTCATTTCATTTTTTGGCGGACCCGTTGTCTCATTTATAAAAATATAAAACCCACGTCGACGACCTGTGTTATAGTCAATATGAAGTTGTACTGTGTTGCTTGTCAGCGAGAGTCCATATCGGGAAGCGT

Amino acid sequence:

MSDINATRLA**IWGIGCDP**CVGDDVTALLTRGEALC

5. *CylK1*

Nucleotide sequence:

ATTCTCTCTCTCAATCACAATGTCTAACATCAATGCCCTCCGTCTCCCTGGCTTTGGTTTTATCCCGTATGCCAGTGGCGACGTCGATTACACTCTCACTCGTGGGGAGAGGTGAGCCCTACATCGAGTGTATCAATGCCCTTATGCGTTGTGTATAGCCTTTCCTGAATACCCCATGCTCAAGGTGTGGCCATCCCACTTCTTAACGGCGATTGTACTGACGTAGATGCAGTAGTCGATGCATATGTTGTTGTTGGCGATATCAGGCTTGGACCATTGAGCCTGCGTCGCAAGTAGGACCCTGTTTGTTCATAATGAAGTATCAGACTAACTTGGTGTGTTCCAGACCACCTACATTCATTTATTTTCTGCTGGTTTTGTTTGTAAACATATAAAAACCCACGTCGACGATCCATTTTGTTACCGCCAATATACTTGTTTTGTGGTATAAGAGTGACGTGACAAGCGAACATGTGTCACATAAATGTAACACAGGCGC

Amino acid sequence:

MSNINALRLP**GFGFIP**YASGDVDYTLTRGESLS

6. *CylK2*

Nucleotide sequence:

CTAAACTCGAGTTCTACATCCTCTTGATCACAATGTCTGATATCAATGCCACCCGTTTCCCAGGCAAGGTCAACCCTCCGTACGTCGGCGATGACGTTGATGATATTATCATTCGTGGCGAGAAGTGAGTCCAGCATCCGTCTGATGATGTACCAGTGGACTCATGGCTATGAATTAGGCTTTGCTGAATACCCGCAAGTCCACTTAATAATAACAGCAAGGTAAACTGTACTGACCTAGACAGAGTGACCGCCGGGGATGACAATGCTAGGCTTGGACCGCTGAGCCTGCATCAGAAGTGGGGCCTTAATTTTGTGGGGAAGCACTGGCTGACAATTCTTTTCCCAGACGACTCACTTCCTCATTTTCTGTGGATCCGTGTTCTTACATTTGTAATCTGATAAAACCCAC

Amino acid sequence:

MSDINATRFP**GKVNPP**YVGDDVDDIIIRGEKLC

7. *AmPOPB*

Nucleotide sequence:

ATGCTACCCACACCATGGGATCCTCACAGTTATCCTCCCACTCGTCGTTCTGACCACGTCGATATCTATCAGAGTGCATCTAGAGGTGAAGTAACAGTACCGGATCCATACCAATGGTTGGAAGAAAATTCAAATGAAGTAGACGAATGGACGACGGCGCAGACAGCTTTCACGCAAGCCTATATTGATAAGAACGCGGATAGGCAGAAGCTCGAGGAGAAAATACGTGCGAGCAAGGACTACGTCAAGGTGATCGATGATCGATGATCAATACATCGTTCTATTTGTGCTGAAAACTTCGTTCATAGTTTTCTGCGCCAACTTTGCTTGATAGCGGGTACTGGTATTGGTTCTATAATAGCGGCCTGCAATCGCAAGCAGGTGTGCAACTTATCTGTCTCTATCAATGCCGAATTCAGACTTGTGCAGTCCTTTACCGCTCCAAGAAACCTGGGCTTCCTGATTTTTCAAAGGGAGACAATGAAGTCGGCGAAGTATTCTTTGATGTAGGGATTTCCACGACATTCGAAATGTTCCTTTGACTTCACTCCTGAAAGCCGAACGTACTCTCTGCTGACGGAACCGCAATTATGGGCATGTGTCGATTCTCCCCTTCTGGCGAGTATTTCGCATATGCAGTGTCCCACTTGGTGAATCGTGTTCCTACATGGCCAACTGCTTGGTCTCATTTTTTGCACAGGGAATCGATTATTTTACTGTCTACGTTCGCCCCACAAGTTCGTCATTGTTTCAGGCACAGACGCCAGCTGAAGGCGGGGACGGCCGATTATCGGATGAAGTGAAATGGTGCAAGTTTACAGCTATAACGTGGACGAAGGACTCCAAAGGTTTTCTTTACCAGGTATAATGCAACCACTAGATCATCAATTCGTTAACTTGCGTCATACAGCGCTTCCCTGCCCAGGAATCTATTGCGGCGAAAGGTCCTGTTAGAGATGCTATGATATGCTACCACAAAGTTGGAACGCCTCAAGGCAGGAATTACTTAGCATTCTGACATTCCCCAAGCTAACTCAGCAGCGCAGTGGAAGACATCATTGTCCAGCAAGACAAGGAGAACCCAGACTGGACATATGGGACCGAGGCGTCAGAGGACGGCAAATATATCTATCTAGTGGTATACAAGGATACCTCGAAGGCAAGGCTCCAATTTTCATTTCCCGACGTCAATAACCTCCATACCACCAGCAAAATCTCCTATGGGTTGCAGAATTCGATAAGGATGGGATCAAGCCGGAAATTCCCTGGCGGAAAGTCATCAATGAATATGTGGCAGATTACCTTGTGTGAGTCCTGTCCTGCTCCAGATCCTCTTTATAACTCGGAATGGTATAGTATCACGAACCACGGACCTTTGATCTATGTCAAGACTAACCTGAATGCGCCCCAATATAAGGTTGTCACTATCGACCTTTCGACAGGCGAACCCGAAATTCGTGATTTCATTCCGGAACAGAAAGATGCGAAGCTCACTCAGGTCAAATGTGTCAACAAGGAGTATTTTGTCACCATCTACAAGCGCAATGTATTTTCATTTATTTTGATTTTGAATTTTTCTAACGCCGATAATGCACAGGTCAAAGACGAAATATATCTTTACTCCAAGGCAGGCGTTCAACTTACTCGTCTAGCGTCAGACTTTGTTGGCGTTGCATCTGTAACGAACAGAGAGAAACAACCGCATTTCTTCCTTACGTTCTCTGGATTTAACACGCCGGGCACTATTTCTTGCTACAATTTTGCAGCTCCAGAGTCACAGCGTCTAAGCATCCTTCGGACGACGAAGATAAATGGACTGAATCCAGATGACTTCGAGAGCACACAAGTCTGGTATGAAAGCAAAGATGGAATGAAAGTTCCAATGTTCATCGTTCGTCACAAATCAACGAAATTTGATGGGACGGCCGCGGCTATTCAAAACGGTAATCCTTCCTCCCCTTTCAGACCAAATTTTGATTTGATTTGCGCAGGTTATGGCGGTTTCGCGATTACTGCTGATCCATTCTTTAGTCCCATCATCCTCACCTTTATGCAAACTTATGGCGCAATCCTGGCTGTCCCGAACATCAGAGGTGGAGGCGAATTCGGTGGAGAATGGCACAGGGCCGGAAGACGAGAAAATAAGGTTTGTACCCATCGCTTTCTATTCCTGATTCAGCCTGGACCTCTGCGATAGGGAAATACTTTTGATGATTTCATCGCTGCCGCGTATGTCTGCCGCTGTTCAATTCGTGATTTCACAGGCTCAACCGTTAAAGTCAATTTCTCGTCAGAAACAAGTACGCAGCTCCAGGCAAGGTGGCCATCACTGGTGCATCAAACGGCGGTAGAGTAACCCTCGTTCTTATTTTCATCCAAGTACTCACCTTGCAACGTTAAAATAGGTTTTCTTGTTTGTGGTTCCATAGTTAGGGCGCCAGAGGGAACATTCGGTGCTGCAATTGCCGAAGGTGGTGTCGCGGACCTCCTAAAGGTAATTTTGTTGTCCACAATATCCTTCCGCGCTCTCTAATTTCTGCTCCCGAGTTCAATAAATTCACCGGGGGTGAGCTGATGTGGGTCTTGTCCATTGTTGATTTGATTAAATACATCGTCAGCGATGGCGTGGACGAGTGAATATGGAAATCCTTCCAATAAGGAAGACTTTGACTTTGTCGAAGCATTGTCCCCCGTACATAACATACCCAAGGACAGGGTCCTTCCAGCCACATTACTTATGATTAATGCAGGTGGGTGACAATACTGAGCCGAGATTTAGCAATACCTAATGCTAGGCTGTCATCAGGTGACGACCGTGTAGTTCCAATGCATTCCCTCAAGTTCATCGCAAGGCTTCAGCACAATGTGCCTCACAATCCCTATCCATTGTTAATCCGTGTGGATAAATCTTGGCTTGGTCATGGTTTTGGGAAGACAACAGACAAACAGTAAATCGCCACCTTTCTACGCTATTCCATTACTTATATTATCCAGTACCAAAGATGCTGCTACTAAGTGGGGATTCGTAGCACAGTCTTTAGGGCTGGAATGGAAAACGGGTTGA

Amino acid sequence:

MLPTPWDPHSYPPTRRSDHVDIYQSASRGEVTVPDPYQWLEENSNEVDEWTTAQTAFTQAYIDKNADRQKLEEKIRASKDYVKFSAPTLLDSGYWYWFYNSGLQSQAVLYRSKKPGLPDFSKGDNEVGEVFFDPNVLSADGTAIMGMCRFSPSGEYFAYAVSHLGIDYFTVYVRPTSSSLFQAQTPAEGGDGRLSDEVKWCKFTAITWTKDSKGFLYQRFPAQESIAAKGPVRDAMICYHKVGTPQVEDIIVQQDKENPDWTYGTEASEDGKYIYLVVYKDTSKQNLLWVAEFDKDGIKPEIPWRKVINEYVADYLVITNHGPLIYVKTNLNAPQYKVVTIDLSTGEPEIRDFIPEQKDAKLTQVKCVNKEYFVTIYKRNVKDEIYLYSKAGVQLTRLASDFVGVASVTNREKQPHFFLTFSGFNTPGTISCYNFAAPESQRLSILRTTKINGLNPDDFESTQVWYESKDGMKVPMFIVRHKSTKFDGTAAAIQNGYGGFAITADPFFSPIILTFMQTYGAILAVPNIRGGGEFGGEWHRAGRRENKGNTFDDFIAAAQFLVRNKYAAPGKVAITGASNGGFLVCGSIVRAPEGTFGAAIAEGGVADLLKFNKFTGAMAWTSEYGNPSNKEDFDFVEALSPVHNIPKDRVLPATLLMINAGDDRVVPMHSLKFIARLQHNVPHNPYPLLIRVDKSWLGHGFGKTTDKHTKDAATKWGFVAQSLGLEWKTG
