# Supplementary material for: Differential Expression of Amanitin Biosynthetic Genes and Novel Cyclic Peptides in Amanita molliuscula
Source: J Fungi (Basel). 2021 May 14;7(5):384. doi: 10.3390/jof7050384 (PMC8156247; doi:10.3390/jof7050384)
Supplement: Supplementary file 1 [file jof-07-00384-s001.zip › supplementary files/Supplemetary file 2.docx]

Supplementary file 2. Genomic DNA and amino acid sequences of amanitin biosynthetic genes (introns underlined, and start and stop codons in red):

1. *AmAMA1*

Nucleotide sequence:

CCTCTTAAAGCTCCCAAATCACAATGTCTGACATCAATGCCACCCGTCTTCCCATCTGGGGAATCGGCTGTAACCCCTGCGTCGGTGACGACGTCACTACACTCCTCACCCGGGGCGAGGCGTAAGCAGAGTCTCTCTACAATAATGTACCAATGCACTTATGCCTTACATGTTAGCCTTTGCTAAATACCGCTCCAATGGTCCGCTGCTATGACTGGAAGGTATTGATCGCTTCATAACGACGAAACAAGGCAGTTGCACTGACTTAGTAGTGGACGAACAAGTTGTCGACGTTATCAGGCTTGGATCGTTGAGTCTGCGTCGGAAGTAGGACCTTTCCGTTTTGTGGCAAAACACGAGGCTAAATTGTCTTTTCTCAGACAACTCACATTCTCCTTTTTTCTGTTGGCGGATCCGTTGTCTCATTTGTAAAAATATAAAACCCACGTCGATGATCTGTGTTGTAGTCAGAATAAAGTTGTACTTTTGCCATGC

Amino acid sequence:

MSDINATRLPIWGIGCNPCVGDDVTTLLTRGEALC

2. *AmAMA2-1*

Nucleotide sequence:

CCTCTAAAGCTCCCAAACCACAATGTCTGACATCAATGCCACCCGTCTTGCCATCTGGGGAATCGGCTGTAACCCGTGCGTCGGTGACGATGTCACTGCACTCCTCACCCGCGGCGAGGCGTAAGCAGAATTTCTCTTAAATAATGTACCAATGCACTTATGCGTCGCGTATCAGTCTTTGCTAAATACGCAATCCATTGACCCGCTGCTATGACACGGAGGTATTATCATCTCACTTATTAACGATGATACAAGGCAGAAGTTGATTCAAACGTAGTAGTGGGCGATACAAGTTGTTGATCTTATCAGGCTTGGACCGTTGAGCCTGCGTCGGAAGTAGGCCCTTCTTGTTTCTGGAAAAACACAAGGCTAAATCGTCTTTTCTCAGACAACTTTCATTTCATTTTTTGGCGGACCCGTTGTCTCATTTATAAAAATATAAAACCCACGTCGATGACCTGTGTTATAGTCAATATGAAGTTGTACTGTGTTTCTTGT

Amino acid sequence:

MSDINATRLAIWGIGCNPCVGDDVTALLTRGEALC

3. *AmAMA2-2*

Nucleotide sequence:

CCTCTAAAGCTCCCAAACCACAATGTCTGACATCAATGCCACCCGTCTTGCCATCTGGGGAATCGGCTGTAACCCGTGCGTCGGTGACGATGTCACTGCACTCCTCACCCGCGGCGAGGCGTAAGCAGAATTTCTCTTAAATAATGTACCAATGCACTTATGCGTCGCGTATCAGTCTTTGCTAAATACGCAATCCATTGACCCGCTGCTATGACACGGAGGTATTATCATCTCACTTATTAACGATGATACAAGGCAGAAGTTGATTCAAACGTAGTAGTGGGCGATACAAGTTGTTGATCTTATCAGGCTTGGACCGTTGAGCCTGCGTCGGAAGTAGGCCCTTCTTGTTTCTGGAAAAACACAAGGCTAAATCGTCTTTTCTCAGACAACTTTCATTTCATTTTTTGGCGGACCCGTTGTCTCATTTTATAAAAATATAAAACCCACGTCGACGATCTGTGTTATAGTCAATATGAAGTTGTACTGTGTTTCTTGT

Amino acid sequence:

MSDINATRLAIWGIGCNPCVGDDVTALLTRGEALC

4. *AmAMA3*

Nucleotide sequence:

TGAACCTCTACGTTCAGCACCCAACTCCCATTCGACCACCTCTAAAGCTCCCAAACCACAATGTCTGACATCAATGCCACCCGTCTTGCCATCTGGGGAATCGGCTGTGACCCGTGCGTCGGTGACGATGTCACTGCACTCCTCACCCGCGGCGAGGCGTAAGCAGAATTTCTCTCCAATAATGTACCAATGCACTTATGCGTCGCGTATTAGTCTTTGCTAAATACGCAATCCATTGACCCGCTGCTATGACAAGGAGGTATTATCATCTCACTTATTAACGATGATACAAGGCAGAAGTTGATTCAAACGTAGTAGTGGGCGATACAAGTTGTTGATCTTATCAGGCTTGGACCGTTAAGCCTGCGTCGGAAGTAGGCCCTTCTTGTTTCTGGAAAAACACAAGGCTAAATCGTCTTTTCTCAGACAACTTTCATTTCATTTTTTGGCGGACCCGTTGTCTCATTTATAAAAATATAAAACCCACGTCGACGACCTGTGTTATAGTCAATATGAAGTTGTACTGTGTTGCTTGTCAGCGAGAGTCCATATCGGGAAGCGT

Amino acid sequence:

MSDINATRLAIWGIGCDPCVGDDVTALLTRGEALC

5. *CylK1*

Nucleotide sequence:

ATTCTCTCTCTCAATCACAATGTCTAACATCAATGCCCTCCGTCTCCCTGGCTTTGGTTTTATCCCGTATGCCAGTGGCGACGTCGATTACACTCTCACTCGTGGGGAGAGGTGAGCCCTACATCGAGTGTATCAATGCCCTTATGCGTTGTGTATAGCCTTTCCTGAATACCCCATGCTCAAGGTGTGGCCATCCCACTTCTTAACGGCGATTGTACTGACGTAGATGCAGTAGTCGATGCATATGTTGTTGTTGGCGATATCAGGCTTGGACCATTGAGCCTGCGTCGCAAGTAGGACCCTGTTTGTTCATAATGAAGTATCAGACTAACTTGGTGTGTTCCAGACCACCTACATTCATTTATTTTCTGCTGGTTTTGTTTGTAAACATATAAAAACCCACGTCGACGATCCATTTTGTTACCGCCAATATACTTGTTTTGTGGTATAAGAGTGACGTGACAAGCGAACATGTGTCACATAAATGTAACACAGGCGC

Amino acid sequence:

MSNINALRLPGFGFIPYASGDVDYTLTRGESLS

6. *CylK2*

Nucleotide sequence:

CTAAACTCGAGTTCTACATCCTCTTGATCACAATGTCTGATATCAATGCCACCCGTTTCCCAGGCAAGGTCAACCCTCCGTACGTCGGCGATGACGTTGATGATATTATCATTCGTGGCGAGAAGTGAGTCCAGCATCCGTCTGATGATGTACCAGTGGACTCATGGCTATGAATTAGGCTTTGCTGAATACCCGCAAGTCCACTTAATAATAACAGCAAGGTAAACTGTACTGACCTAGACAGAGTGACCGCCGGGGATGACAATGCTAGGCTTGGACCGCTGAGCCTGCATCAGAAGTGGGGCCTTAATTTTGTGGGGAAGCACTGGCTGACAATTCTTTTCCCAGACGACTCACTTCCTCATTTTCTGTGGATCCGTGTTCTTACATTTGTAATCTGATAAAACCCAC

Amino acid sequence:

MSDINATRFPGKVNPPYVGDDVDDIIIRGEKLC

7. *AmPOPB*

Nucleotide sequence:

ATGCTACCCACACCATGGGATCCTCACAGTTATCCTCCCACTCGTCGTTCTGACCACGTCGATATCTATCAGAGTGCATCTAGAGGTGAAGTAACAGTACCGGATCCATACCAATGGTTGGAAGAAAATTCAAATGAAGTAGACGAATGGACGACGGCGCAGACAGCTTTCACGCAAGCCTATATTGATAAGAACGCGGATAGGCAGAAGCTCGAGGAGAAAATACGTGCGAGCAAGGACTACGTCAAGGTGATCGATGATCGATGATCAATACATCGTTCTATTTGTGCTGAAAACTTCGTTCATAGTTTTCTGCGCCAACTTTGCTTGATAGCGGGTACTGGTATTGGTTCTATAATAGCGGCCTGCAATCGCAAGCAGGTGTGCAACTTATCTGTCTCTATCAATGCCGAATTCAGACTTGTGCAGTCCTTTACCGCTCCAAGAAACCTGGGCTTCCTGATTTTTCAAAGGGAGACAATGAAGTCGGCGAAGTATTCTTTGATGTAGGGATTTCCACGACATTCGAAATGTTCCTTTGACTTCACTCCTGAAAGCCGAACGTACTCTCTGCTGACGGAACCGCAATTATGGGCATGTGTCGATTCTCCCCTTCTGGCGAGTATTTCGCATATGCAGTGTCCCACTTGGTGAATCGTGTTCCTACATGGCCAACTGCTTGGTCTCATTTTTTGCACAGGGAATCGATTATTTTACTGTCTACGTTCGCCCCACAAGTTCGTCATTGTTTCAGGCACAGACGCCAGCTGAAGGCGGGGACGGCCGATTATCGGATGAAGTGAAATGGTGCAAGTTTACAGCTATAACGTGGACGAAGGACTCCAAAGGTTTTCTTTACCAGGTATAATGCAACCACTAGATCATCAATTCGTTAACTTGCGTCATACAGCGCTTCCCTGCCCAGGAATCTATTGCGGCGAAAGGTCCTGTTAGAGATGCTATGATATGCTACCACAAAGTTGGAACGCCTCAAGGCAGGAATTACTTAGCATTCTGACATTCCCCAAGCTAACTCAGCAGCGCAGTGGAAGACATCATTGTCCAGCAAGACAAGGAGAACCCAGACTGGACATATGGGACCGAGGCGTCAGAGGACGGCAAATATATCTATCTAGTGGTATACAAGGATACCTCGAAGGCAAGGCTCCAATTTTCATTTCCCGACGTCAATAACCTCCATACCACCAGCAAAATCTCCTATGGGTTGCAGAATTCGATAAGGATGGGATCAAGCCGGAAATTCCCTGGCGGAAAGTCATCAATGAATATGTGGCAGATTACCTTGTGTGAGTCCTGTCCTGCTCCAGATCCTCTTTATAACTCGGAATGGTATAGTATCACGAACCACGGACCTTTGATCTATGTCAAGACTAACCTGAATGCGCCCCAATATAAGGTTGTCACTATCGACCTTTCGACAGGCGAACCCGAAATTCGTGATTTCATTCCGGAACAGAAAGATGCGAAGCTCACTCAGGTCAAATGTGTCAACAAGGAGTATTTTGTCACCATCTACAAGCGCAATGTATTTTCATTTATTTTGATTTTGAATTTTTCTAACGCCGATAATGCACAGGTCAAAGACGAAATATATCTTTACTCCAAGGCAGGCGTTCAACTTACTCGTCTAGCGTCAGACTTTGTTGGCGTTGCATCTGTAACGAACAGAGAGAAACAACCGCATTTCTTCCTTACGTTCTCTGGATTTAACACGCCGGGCACTATTTCTTGCTACAATTTTGCAGCTCCAGAGTCACAGCGTCTAAGCATCCTTCGGACGACGAAGATAAATGGACTGAATCCAGATGACTTCGAGAGCACACAAGTCTGGTATGAAAGCAAAGATGGAATGAAAGTTCCAATGTTCATCGTTCGTCACAAATCAACGAAATTTGATGGGACGGCCGCGGCTATTCAAAACGGTAATCCTTCCTCCCCTTTCAGACCAAATTTTGATTTGATTTGCGCAGGTTATGGCGGTTTCGCGATTACTGCTGATCCATTCTTTAGTCCCATCATCCTCACCTTTATGCAAACTTATGGCGCAATCCTGGCTGTCCCGAACATCAGAGGTGGAGGCGAATTCGGTGGAGAATGGCACAGGGCCGGAAGACGAGAAAATAAGGTTTGTACCCATCGCTTTCTATTCCTGATTCAGCCTGGACCTCTGCGATAGGGAAATACTTTTGATGATTTCATCGCTGCCGCGTATGTCTGCCGCTGTTCAATTCGTGATTTCACAGGCTCAACCGTTAAAGTCAATTTCTCGTCAGAAACAAGTACGCAGCTCCAGGCAAGGTGGCCATCACTGGTGCATCAAACGGCGGTAGAGTAACCCTCGTTCTTATTTTCATCCAAGTACTCACCTTGCAACGTTAAAATAGGTTTTCTTGTTTGTGGTTCCATAGTTAGGGCGCCAGAGGGAACATTCGGTGCTGCAATTGCCGAAGGTGGTGTCGCGGACCTCCTAAAGGTAATTTTGTTGTCCACAATATCCTTCCGCGCTCTCTAATTTCTGCTCCCGAGTTCAATAAATTCACCGGGGGTGAGCTGATGTGGGTCTTGTCCATTGTTGATTTGATTAAATACATCGTCAGCGATGGCGTGGACGAGTGAATATGGAAATCCTTCCAATAAGGAAGACTTTGACTTTGTCGAAGCATTGTCCCCCGTACATAACATACCCAAGGACAGGGTCCTTCCAGCCACATTACTTATGATTAATGCAGGTGGGTGACAATACTGAGCCGAGATTTAGCAATACCTAATGCTAGGCTGTCATCAGGTGACGACCGTGTAGTTCCAATGCATTCCCTCAAGTTCATCGCAAGGCTTCAGCACAATGTGCCTCACAATCCCTATCCATTGTTAATCCGTGTGGATAAATCTTGGCTTGGTCATGGTTTTGGGAAGACAACAGACAAACAGTAAATCGCCACCTTTCTACGCTATTCCATTACTTATATTATCCAGTACCAAAGATGCTGCTACTAAGTGGGGATTCGTAGCACAGTCTTTAGGGCTGGAATGGAAAACGGGTTGA

Amino acid sequence:

MLPTPWDPHSYPPTRRSDHVDIYQSASRGEVTVPDPYQWLEENSNEVDEWTTAQTAFTQAYIDKNADRQKLEEKIRASKDYVKFSAPTLLDSGYWYWFYNSGLQSQAVLYRSKKPGLPDFSKGDNEVGEVFFDPNVLSADGTAIMGMCRFSPSGEYFAYAVSHLGIDYFTVYVRPTSSSLFQAQTPAEGGDGRLSDEVKWCKFTAITWTKDSKGFLYQRFPAQESIAAKGPVRDAMICYHKVGTPQVEDIIVQQDKENPDWTYGTEASEDGKYIYLVVYKDTSKQNLLWVAEFDKDGIKPEIPWRKVINEYVADYLVITNHGPLIYVKTNLNAPQYKVVTIDLSTGEPEIRDFIPEQKDAKLTQVKCVNKEYFVTIYKRNVKDEIYLYSKAGVQLTRLASDFVGVASVTNREKQPHFFLTFSGFNTPGTISCYNFAAPESQRLSILRTTKINGLNPDDFESTQVWYESKDGMKVPMFIVRHKSTKFDGTAAAIQNGYGGFAITADPFFSPIILTFMQTYGAILAVPNIRGGGEFGGEWHRAGRRENKGNTFDDFIAAAQFLVRNKYAAPGKVAITGASNGGFLVCGSIVRAPEGTFGAAIAEGGVADLLKFNKFTGAMAWTSEYGNPSNKEDFDFVEALSPVHNIPKDRVLPATLLMINAGDDRVVPMHSLKFIARLQHNVPHNPYPLLIRVDKSWLGHGFGKTTDKHTKDAATKWGFVAQSLGLEWKTG
