# Supplementary figures and images for: Differential Expression of Amanitin Biosynthetic Genes and Novel Cyclic Peptides in Amanita molliuscula
Source: J Fungi (Basel). 2021 May 14;7(5):384. doi: 10.3390/jof7050384 (PMC8156247; doi:10.3390/jof7050384)

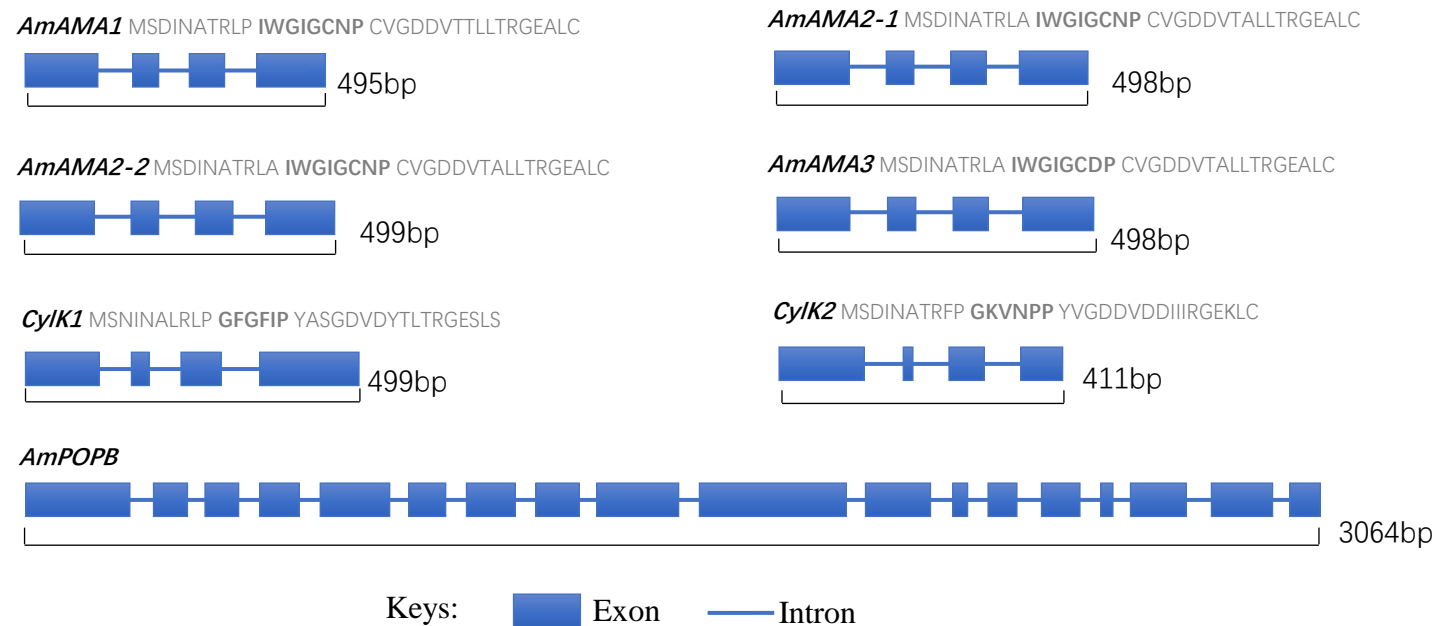

Fig. S1 Gene structure of amanitin biosynthetic genes in *Amanita molliuscula*.

Supplement: Supplementary file 1 [file jof-07-00384-s001.zip › supplementary files/Figure S1.pdf]
